# The Efficacy and Safety of Direct Oral Anticoagulants Compared to Warfarin for Left Ventricular Thrombus Resolution

**DOI:** 10.3390/jcm14062129

**Published:** 2025-03-20

**Authors:** Mariana Sousa Paiva, Francisco Gama, Samuel Azevedo, Pedro M. Lopes, Francisco Albuquerque, Carla Reis, Pedro Freitas, Sara Guerreiro, João Abecasis, Marisa Trabulo, António M. Ferreira, Regina Ribeiras, Jorge Ferreira, Pedro Pulido Adragão

**Affiliations:** 1 Department of Cardiology, Hospital Santa Cruz, Unidade Local de Saúde Lisboa Ocidental, 2790-134 Carnaxide, Portugal; 2 Hospital da Luz Saúde, 1500-650 Lisbon, Portugal; 3 Hospital Lusíadas Lisboa, 1500-458 Lisbon, Portugal

**Keywords:** intraventricular thrombus, warfarin, direct oral anticoagulants, bleeding, systemic arterial embolism

## Abstract

**Background and Aim:** Left ventricular thrombus (LVT) is a common complication of myocardial infarction (MI) and heart failure with reduced ejection fraction (HFrEF), typically managed with vitamin K antagonists (VKAs) for up to six months. However, data on direct oral anticoagulants (DOACs) for LVT treatment remain limited and conflicting. This study evaluates the effectiveness and safety of DOACs compared to warfarin for LVT resolution. **Methods**: We conducted a single-center retrospective cohort study of consecutive patients diagnosed with LVT from January 2010 to May 2024. The primary outcome was LVT resolution at 24 months. Safety outcomes included major bleeding and thromboembolic events. Diagnosis and follow-up were performed via echocardiography, with cardiac magnetic resonance and computed tomography as needed. Anticoagulant type, dose, duration, and concurrent antiplatelet therapy were at the treating physician’s discretion. **Results**: Among 171 patients (82.5% male, mean age 59.8 ± 14.7 years), 99 received DOACs and 72 received warfarin. LVT resolution was higher with DOACs (66.7% vs. 50%, HR 2.0, 95% CI 1.07–3.73, *p* = 0.029), with a trend toward faster thrombus resolution (185 vs. 220 days, *p* = 0.214) though statistically not significant. DOAC use remained an independent predictor of LVT resolution, regardless of antiplatelet use. Major bleeding (2.9%), thromboembolic events (5.3%), and mortality (5.3%) were similar between groups. **Conclusions**: DOAC therapy was associated with higher LVT resolution rates and a comparable safety profile to warfarin. Further randomized clinical trials are warranted to validate these findings.

## 1. Introduction

Even though left ventricular thrombus (LVT) incidence has been decreasing in recent years due to prompt primary percutaneous coronary intervention (PCI) and improved overall periprocedural care after acute myocardial infarction (MI), it still represents a frequently recognized complication of heart failure, mainly due to heightened sensitivity of contemporaneous imaging modalities [1,2]. Major predisposing factors for thrombus formation are hypercoagulability, endothelial lesion, and blood stasis (i.e., Virchow triad) while its most fearful complication is systemic thromboembolism, such as ischemic stroke [3].

Current European guidelines recommend anticoagulation up to 6 months guided by repeated imaging evaluation, whereas American guidelines endorse only vitamin K antagonists (VKAs) with a target international normalized ratio (INR) of 2.0–3.0 as the treatment of choice [4,5]. DOACs are an attractive alternative to VKAs due to their favorable dosing convenience and have demonstrated superior efficacy and safety in patients with non-valvular atrial fibrillation. In the setting of acute venous thromboembolism, they showed non-inferior efficacy but an overall superior safety profile [6]. Nevertheless, contrasting to thromboembolic prophylaxis in atrial fibrillation, clinical evidence regarding comparison between DOACs and VKAs in patients with LVT remains limited to a few retrospective cohort studies with contradicting results and four small-sample randomized trials [7,8,9,10].

Our aim is to describe the effectiveness and safety of DOACs for the treatment of LVT versus warfarin in a single center for an unselected population with MI and/or HFrEF.

## 2. Methods

### 2.1. Study Design

This was a single-center retrospective cohort study of consecutive patients with recently diagnosed LVT.

We used the STROBE cohort checklist when writing our manuscript [11].

This study was carried out in compliance with the amended Declaration of Helsinki. The requirement for patients’ informed consent was waived under the framework of the National Health Authority’s Quality Certification Program for the purpose of conducting an internal audit.

### 2.2. Participants

Patients were aged over 18 years with LVT diagnosis between 1 January 2010 and 31 May 2024.

Patients were excluded from the study if they were treated with non-oral anticoagulants, such as low-molecular-weight heparin (LMWH) or unfractionated heparin (UFH), or if they declined anticoagulation altogether. Additional exclusion criteria included having less than 60 days of follow-up, lack of imaging reassessment of the ventricular thrombus, prior anticoagulation before the diagnosis, being treated at other hospitals, submission to heart transplantation (HTx) or left ventricular assist device (LVAD) implantation before re-evaluation, death within 30 days of diagnosis, and incomplete data records. Patients with severe hepatic and renal insufficiency were excluded from this study. Specifically, exclusion criteria included a creatinine clearance < 30 mL/min/1.73 m^2^ and significant hepatic impairment, defined as alanine aminotransferase or aspartate aminotransferase levels > 3 times the upper limit of normal or total bilirubin > 2 times the upper limit of normal.

Inclusion time among those with advanced HF was terminated if heart transplant (HTx) was performed. Decisions regarding the type, dose, and duration of anticoagulation and any concomitant antiplatelet therapy were left to the physician’s judgement.

Direct oral anticoagulation (i.e., apixaban, dabigatran, edoxaban, or rivaroxaban) dosages were in agreement with European labels for atrial fibrillation thromboprophylaxis. Apixaban 5 mg b.i.d. (2.5 mg b.i.d. with at least two of three criteria: age ≥ 80 years, body weight ≤ 60 Kg, serum creatinine ≥ 1.5 mg/dL), dabigatran 150 mg b.i.d. (110 mg b.i.d. in patients ≥ 80 years, increased bleeding risk, or concomitant use of verapamil), edoxaban 60 mg o.d. (30 mg o.d. with any of the following: CrCl 15–50 mL/min, body weight ≤ 60 Kg, concomitant use of ketoconazole, erythromycin, ciclosporin, dronedarone), and rivaroxaban 20 mg o.d. (15 mg o.d. in patients with eGFR 30–49 mL/min/1.73 m^2^) were used.

Triple therapy was defined as dual antiplatelet therapy (DAPT) in addition to oral anticoagulation. According to ESC guidelines, no potent P2Y12 inhibitors (i.e., ticagrelor or prasugrel) were used with concomitant oral anticoagulation.

### 2.3. Imaging Assessment

Thrombus diagnosis and subsequent assessments were performed with echocardiography and complemented with cardiac magnetic resonance (CMR) or cardiac computed tomography (CT), when appropriate. Whenever the acoustic window was suboptimal or findings were inconclusive, echocardiograms were complemented with intravenous ultrasound contrast agents (microbubbles).

In addition to left ventricular ejection fraction and left ventricular telediastolic indexed volume, we assessed the wall motion severity index (WMSI) using echocardiography. Each of the 17 left ventricular segments was scored according to its motion, with scores ranging from 1 (normal) to 5 (aneurysmal). The WMSI was calculated as the sum of all segment scores divided by the number of segments analyzed.

Timing for imaging assessment was left to the clinician’s decision. Echocardiography findings were independently interpreted by two expert cardiologists (JA, FG), blinded to the oral anticoagulant patient regimen. All included patients had a follow-up scan to document thrombus evolution.

### 2.4. Endpoints

The primary endpoint was left ventricular thrombus resolution. Secondary endpoints were all-cause mortality, ischemic stroke/transient ischemic attack, peripheral embolism, and hemorrhagic events. Bleeding episodes were classified according to location (i.e., epistaxis, intracranial, digestive, retroperitoneal, genito-urinary) and severity (assessed by the Bleeding Academic Research Consortium (BARC) classification) [12].

Event assessment was performed using electronic hospital records and telephonic consulting when appropriate. These events were adjudicated by 5 independent physicians (MSP, SA, FG, PL, FA) who were blinded to each patient’s treatment.

### 2.5. Statistical Analysis

Continuous variables are described as mean ± standard deviation (SD) or as median (interquartile range), while categorical variables are described as percentages. To compare variables, we used the chi-square test and the Fisher’s exact test as appropriate for categorical variables and the Mann–Whitney test or Student’s *t*-test for continuous variables.

The time to clinical event was portrayed using Kaplan–Meier survival curves and compared with the log-rank test. Clinical, demographic, and echocardiographic variables were evaluated using univariable Cox proportional hazards regression to determine their association with LV thrombus (LVT) resolution. Variables with *p* < 0.05 in the univariable analysis were subsequently included in a stepwise multivariable Cox proportional hazards regression model, where those with *p* < 0.05 were considered statistically significant.

Additionally, we assessed potential interactions between predictors in the multivariable Cox model by including interaction terms. Specifically, we examined whether the effects of certain factors on thrombus resolution were modified by other covariates. Interaction terms were tested by incorporating multiplicative terms (e.g., Variable A × Variable B) into the model, with significance considered at a *p*-value of <0.10, given the exploratory nature of interaction testing.

All other statistical tests were 2-sided and statistical significance was accepted with a *p*-value of <0.05. SPSS statistics software version 25.0 (SPSS, Chicago, IL, USA) was used to perform all statistical evaluations.

## 3. Results

**Figure 1:**
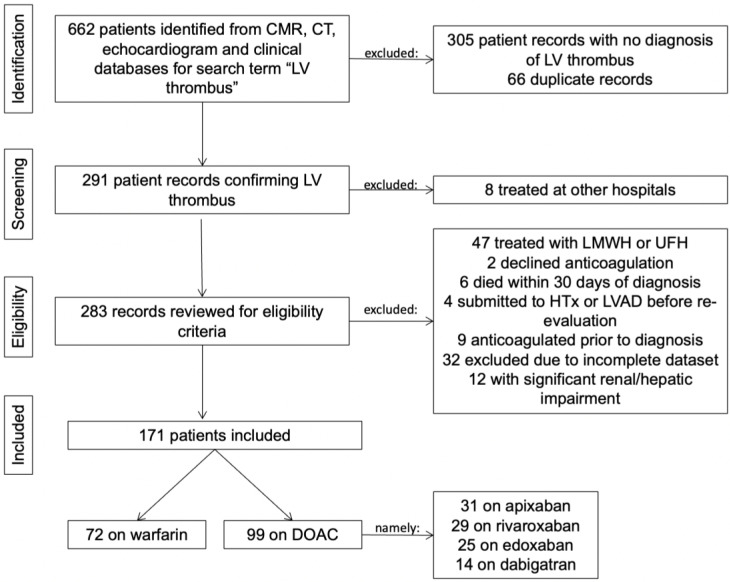
Flowchart of patient selection for the study cohort.

One hundred and seventy-one patients with a confirmed diagnosis of LV thrombus were included (mean age of 59.8 ± 14.7 years), the majority being male (*n* = 141, 82.5%)—Figure 1.

Seventy-two patients (42%) were receiving warfarin. Of those on DOACs (N = 99, 58%), 31 were on apixaban, 29 on rivaroxaban, 25 on edoxaban, and 14 on dabigatran. In the DOAC group, the majority were treated with the full-dose regimen (76 (76.8%) vs. 23 (23.2%) patients), in accordance with the dose regimen used for AF thromboprophylaxis (Supplementary Figure S1).

Baseline characteristics are as follows and shown in Table 1. Patients receiving DOACs were younger (55.4 vs. 65.9 years, *p* < 0.001) and tended to have more cardiovascular risk factors. DOAC patients had higher NT-proBNP levels (2490 vs. 1320 mg/dL, *p* = 0.036) and greater creatinine clearance (119 vs. 89.7 mL/min/1.73 m^2^, *p* = 0.001) compared to those on warfarin.

Atrial fibrillation was more frequent in the DOAC group (24.2% vs. 9.7%, *p* = 0.015) while prior myocardial infarction (MI) had a similar proportion between groups (66 (66.7%) vs. 55 (76.4%), *p* = 0.168).

Patients on DOAC had worse ejection fraction (34 ± 12% vs. 38 ± 11%, *p* = 0.037) and increased diastolic left ventricular dimensions (79 mL/m^2^ vs. 66 mL/m^2^, *p* = 0.007).

The median follow-up period tended to be longer within the warfarin group (1480 days, (interquartile range (IQR): 719–2675) vs. 757 days (IQR: 400–1146), *p* < 0.001).

### 3.1. Primary Endpoint—LVT Resolution

LVT resolution during follow-up occurred in 111 patients (64%) (Table 2) and had a DOAC therapy (70 (70.5%) vs. 41 (56.9%), *p* = 0.063) and antiplatelet therapy association (APT, 42 (77.8%) vs. 69 (59.0%), *p* = 0.001). These patients had significantly lower WMSI at the initial assessment (1.9 ± 0.3 vs. 2.2 ± 0.4, *p* = 0.014).

**Figure 2:**
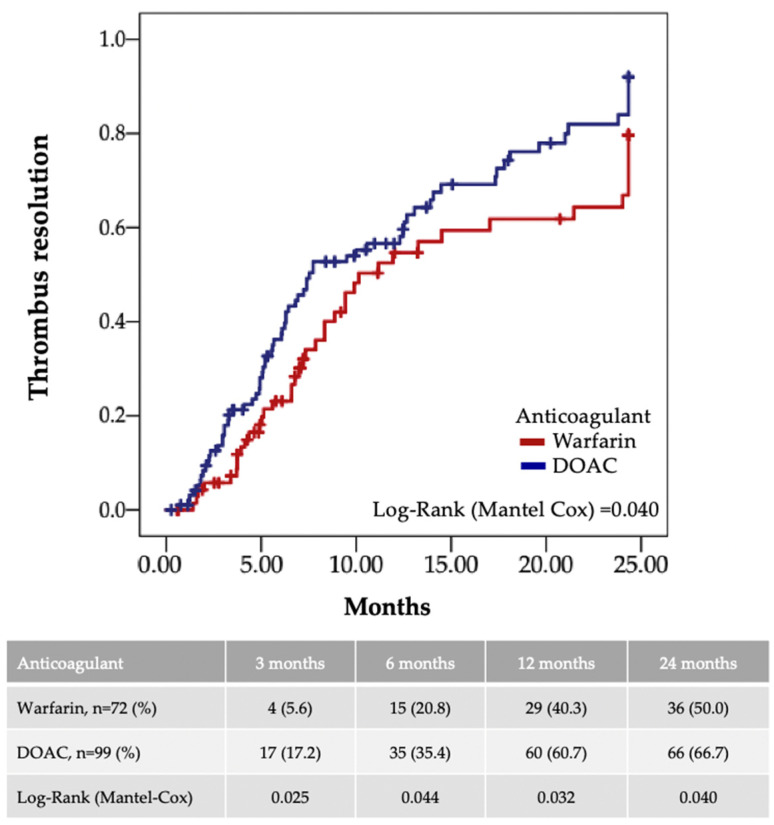
Kaplan–Meier curve for thrombus resolution comparing DOAC and warfarin over 24 months.

Patients on DOACs tended to have faster thrombus resolution (185 days (IQR: 97–377) vs. 220 days (IQR: 128–378), *p* = 0.214). DOAC therapy yielded significantly higher rates of thrombus resolution (Figure 2), evident as early as at 3 months’ follow-up (17.2% in the DOAC group vs. 5.6% in the warfarin group, *p* = 0.025) and persisted through 24 months (66.7% in the DOAC group vs. 50.0% in the warfarin group, *p* = 0.040).

An analysis was conducted to assess whether the efficacy of thrombus resolution differed across the various DOAC types (apixaban, rivaroxaban, edoxaban, dabigatran). However, no significant differences were observed in thrombus resolution rates between the DOAC groups.

Univariate Cox proportional hazard regression analysis yielded DOAC use (compared to warfarin) (HR: 1.69, 95% CI: 1.13–2.53, *p* = 0.011), simultaneous use of antiplatelet therapy (APT) (HR: 1.86, 95% CI: 1.27–2.73, *p* = 0.002), and follow-up WMSI (HR: 0.51, 95% CI: 0.26–0.99, *p* = 0.047) as significant predictors of the LVT resolution.

On multivariate analysis, DOACs remained a significant predictor of outcome independent of age, type of myocardial infarction, simultaneous antiplatelet therapy, and final WMSI (Table 3), HR: 1.9 95% CI: 1.01–3.45, *p* = 0.047. The interaction between DOAC and APT was not statistically significant (*p* = 0.346), suggesting that their effects on thrombus resolution are independent of each other (Supplementary Table S1).

### 3.2. Secondary Endpoints

The incidence of thromboembolic events was nine (5.2%), similar between groups with three (3.0%) events among patients under DOAC and six (8.3%) of those under warfarin therapy (chi-square test *p* = 0.169). All-cause mortality was similar between groups (DOAC 5 (5.1%) vs. VKA 4 (5.6%), *p* = 1.0).

The incidence of bleeding BARC ≥ 3 was also similar under DOAC (n = 1 (1.0%)) and under warfarin (n = 3 (4.2%)) (*p* = 0.311). In addition, the number of blood transfusions was also similar between groups (DOAC 1 (1.0%) vs. VKA 2 (2.8%), *p* = 0.573). One patient under warfarin had a retroperitoneal bleeding. No intracranial hemorrhagic events occurred (Table 4).

## 4. Discussion

**Figure 3:**
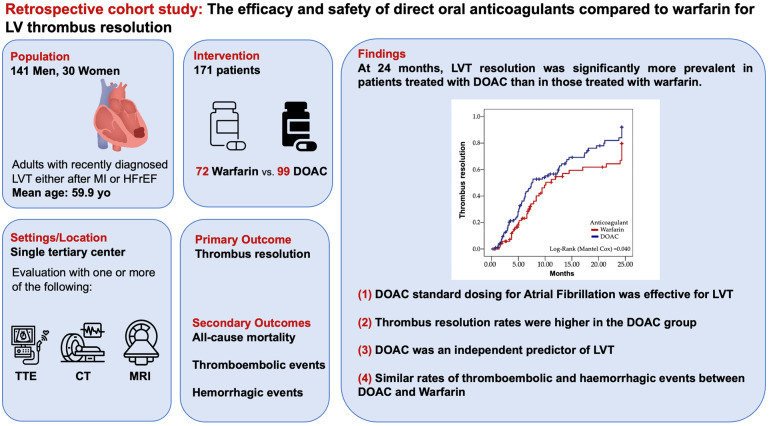
Efficacy and Safety of Direct Oral Anticoagulants (DOACs) Compared to Warfarin for Left Ventricular Thrombus (LVT) Resolution.

The main findings of our study were: (1) DOAC dosage for atrial fibrillation was effective for thrombus resolution; (2) thrombus resolution rates within the DOAC group were consistently higher across different time points, despite a higher prevalence of patients with more dilated left ventricles; (3) DOACs predicted thrombus resolution independently from simultaneous antiplatelet therapy and segmental wall motion changes; (4) both groups tended to have similar thromboembolic and hemorrhagic events (Figure 3).

Atrial fibrillation DOAC dosage for off-label use regarding LVT has been the mainstay therapy for studies focusing on this issue. Results of this therapy are usually displayed according to efficacy for thrombus resolution, bleeding, and thromboembolic events.

To date, our cohort is the second to report a significantly higher number of patients receiving DOACs compared to those on VKAs [13]. This finding can be attributed to two main factors. Firstly, advancements in imaging techniques have led to an increased diagnosis of LVT, prompting a preference for DOACs due to their ease of management. Secondly, a significant number of patients on LMWH and UFH were excluded from our study, as shown in Figure 1, many of whom were further treated with VKAs.

### 4.1. Thrombus Regression

Although current European guidelines recommend both vitamin K antagonists and DOACs for LVT, in our study, only DOACs remained significant for thrombus resolution after adjusted Cox proportional hazards regression model analysis. Time till complete thrombus regression tended to be shorter for those on DOACs.

In keeping with these findings, two recent small sample size RCTs comparing rivaroxaban and apixaban to warfarin in a subset of patients with LVT without AF highlight the efficacy of these two direct factor Xa inhibitors in thrombus disappearance [7,8]. Moreover, similar findings were obtained by Jones DA et al. in a retrospective cohort analysis of LVT following ST elevation myocardial infarction (STEMI) [14]. Furthermore, a recent cohort study from China also examined the effectiveness of DOACs versus warfarin in patients with LV thrombus. While this study found no significant difference in thrombus resolution between the two groups within 90 and 180 days, it suggested that DOACs, such as rivaroxaban and dabigatran, may be a viable alternative to warfarin for LV thrombus management [15]. Additionally, a recent multicenter retrospective study by Al-Abcha et al., which employed propensity-score matching, found no significant difference in thrombus resolution between warfarin and DOACs. Their multivariate analysis reported an odds ratio (OR) of 0.94 (95% confidence interval (CI) 0.858 to 1.029, *p* = 0.18) [16].

A more predictable pharmacodynamic due to fewer drug and food interactions of DOACs yields a consistent therapeutic range of concentrations throughout the day, potentially explaining their improved efficacy for this endpoint [17].

As in previous studies, our analysis showed that, regardless of the therapeutic choice, persistence of LVT remained common in follow-up imaging evaluation. Patients with LVT have significantly bad prognosis with higher rates of major adverse cardiovascular events, major bleeding events, all-cause death, and a tendency towards embolic complications [18]. Alternative therapeutic regimes are needed to improve these clinical endpoints.

Blood stasis and hypercoagulability are two out of three variables depicted in the Virchow triad and both present in heart failure patients with dilated left ventricles and reduced ejection fraction [19]. In our analysis, DOAC therapy was an effective therapy, even considering the higher prevalence of negatively remodeled left ventricles, noticeable by lower ejection fraction and WMSI, and larger chamber sizes. These findings highlight the improved therapeutic effect of this pharmacological class, even on those with worse prognosis and wider areas of stunned myocardium. Furthermore, lower WMSI at follow-up emerged as a significant predictor for thrombus resolution. Being a surrogate for blood stasis, this study stresses the importance of improved contractility as a therapeutic target tackled with intensive disease modifying therapy, either pharmacologically (i.e., ARNi, BB, MRA, SGLT2i) or through revascularization when appropriate [20,21,22].

In the multivariate analysis, DOAC therapy remained an independent predictor of thrombus resolution, even after adjusting for APT and WMSI. This suggests that the efficacy of DOACs in thrombus regression was not merely a result of combined therapeutic strategies but rather an intrinsic effect of this pharmacological class. The absence of a significant interaction between DOAC and APT reinforces the hypothesis that oral direct anticoagulation exerts an independent impact on thrombus burden reduction.

Taking all into consideration, in this study, DOACs were an effective therapy for LVT resolution. Previous studies not only had inconsistent use of follow-up image assessment of thrombus evolution but also a low concordance regarding imaging modality choice between exams [18,19,20]. Such variability probably underpowers the DOAC arm, hindering consistent display of improved efficiency when compared to its VKA alternative.

### 4.2. Safety Endpoints

In our cohort, patients on DOACs had similar rates of bleeding events. These findings are in line with previously published studies that yielded at least non-inferiority in this regard [14,18,19]. As in this study, limited sample size hinders any assertive conclusions on the safety bleeding profile of DOACs for LVT treatment. Additionally, we acknowledge that patients on DOACs were significantly younger, which may have contributed to a lower intrinsic bleeding risk.

Furthermore, the evidence is conflicting regarding thromboembolic events in patients with LVT undergoing DOAC or VKA therapy. In a large retrospective study by Robinson et al. of 514 patients (185 on DOACs vs. 300 on warfarin), with mean follow-up of 351 days and after multivariable analysis, anticoagulation with DOACs (HR: 2.64; 95% CI, 1.28–5.43; *p* = 0.01) was a significant predictor of systemic embolism [23]. On the other hand, another retrospective analysis demonstrated a protective effect of DOACs for thromboembolism [24]. As for our study, and mirroring previous smaller observational analyses, DOAC and warfarin therapy yielded similar thromboembolic event rates [22,25,26,27]. Further studies are needed to enlighten about current discrepancies [28].

### 4.3. Study Limitations

This study has several limitations that should be acknowledged. First, it was a retrospective, single-center analysis, which restricts our ability to draw causal or mechanistic conclusions. Additionally, LVT diagnosis was primarily based on echocardiography, a modality with lower sensitivity and specificity compared to cardiac MRI or CT, potentially affecting diagnostic accuracy modalities [29].

Another important limitation is the lack of centralized INR measurements and individualized dosing adjustments, which prevented an accurate assessment of time in therapeutic range (TTR) for patients on VKAs. This may have influenced treatment efficacy and could be a confounding factor when comparing anticoagulation strategies.

Furthermore, the study’s small sample size limits the generalizability of our findings regarding both primary and secondary endpoints. However, despite this constraint, our study represents the sixth largest comparison of DOACs and VKAs for LVT resolution, providing valuable insights into this clinical question.

It is also important to acknowledge that we did not adjust for factors related to infarction and its consequences, which may have influenced thrombus resolution. Specifically, variables such as optimized medical therapy, revascularization status, infarct type, and the affected vessel were not included in the adjusted models. This limitation is largely due to the retrospective design and the long inclusion period starting in 2010, during which treatment standards evolved significantly. As a result, data on these variables were not consistently available for all patients, restricting our ability to fully assess their influence on thrombus resolution.

## 5. Conclusions

This study highlights the efficacy of DOACs on LVT resolution, with a similar safety profile to warfarin. Left ventricular segmental contractility emerges as an important predictor of both thrombus formation and regression. Further randomized controlled trials are needed to confirm these findings and robustly assess safety endpoints.

### Key Messages

1. LVT is a serious complication often following myocardial infarction or in patients with reduced left ventricular ejection fraction, increasing embolic risk.
2. While warfarin has been the standard treatment, DOACs offer potential benefits, including fixed dosing and fewer drug interactions, though their efficacy and safety in LVT resolution remain unclear.
3. This study compares DOACs and warfarin for LVT resolution, assessing both efficacy (thrombus resolution) and safety (bleeding risks, embolic events), suggesting DOACs may be equally effective with potentially lower bleeding risks.
4. These findings provide real-world evidence to guide clinicians and may support greater use of DOACs.
5. However, randomized controlled trials are needed to confirm the benefits and risks of DOACs in this specific patient population.

## Figures and Tables

**Table 1:** Baseline characteristics of patients with left ventricular thrombus treated with direct oral anticoagulants or warfarin.

	Overall (N = 171)	Warfarin (N = 72)	DOAC (N = 99)	*p*-Value
**Demographics**				
Male, n (%)	141 (82.5)	55 (76.4)	86 (86.9)	0.08
Age, years (mean ± SD)	59.8 ± 14.7	65.9 ± 14.0	55.4 ± 13.7	<0.0001
BSA, m^2^ (median (IQR))	1.88(1.77–2.03)	1.86 (1.73–2.02)	1.89 (1.80–2.04)	0.20
Diabetes, n (%)	30 (17.5)	10 (13.9)	20 (20.2)	0.28
Hypertension, n (%)	114 (66.7)	47 (65.3)	67 (67.7)	0.74
Dyslipidemia, n (%)	97 (56.7)	40 (55.6)	57 (57.6)	0.79
Current smoker, n (%)	45 (26.3)	21 (29.2)	24 (24.2)	0.49
**Laboratory findings**				
NTproBNP, mg/dL (median (IQR))	2272 (747–4898)	1320 (488–4087)	2490 (954–6602)	0.04
Clearance, mL/min/1.73 m^2^ (mean ± SD)	106.3 ± 57.4	89.7 ± 35.6	119 ± 67.1	0.001
**Atrial fibrillation, n (%)**	31 (18.1)	7 (9.7)	24 (24.2)	0.02
**Etiology**				
Myocardial infarction, n (%)	121 (70.8)	55 (76.4)	66 (66.7)	0.17
STEMI,	71 (41.5)	26 (36.2)	45 (45.5%)	0.22
HFrEF, n (%)	102 (59.6)	31 (43.1)	71 (71.7)	<0.0001
**Baseline echo findings**				
Ejection fraction, % (mean ± SD)	36 ± 11	38 ± 11	34 ± 12	0.04
TDVi, mL/m^2^ (median(IQR))	73.0 (57.4–96.8)	66.0 (52.0–83.0)	79.3 (62.3–107.3)	0.007
WMSI, n (mean ± SD)	2.1 ± 0.4	2.0 ± 0.4	2.1 ± 0.4	0.10
Follow-up period, days (median (IQR))	934 (436–1536)	1480 (719–2675)	757 (400–1146)	<0.001

*p*-values were calculated using the chi-square test for categorical variables and the Mann–Whitney test or Student’s *t*-test for continuous variables.

**Table 2:** Baseline characteristics of the study population stratified by thrombus resolution status.

	Overall (N = 171)	Thrombus Maintenance (N = 60)	Thrombus Resolution (N = 111)	*p*-Value
**VKA, n (%)**	72 (42.1)	31 (51.7)	41 (37.0)	0.06
**DOAC, n (%)**	99 (57.9)	29 (48.3)	70 (63.0)	0.06
Edoxaban, n (%)	25 (14.6)	5 (8.3)	20 (18.0)	0.64
Apixaban, n (%)	31 (18.1)	9 (15.0)	22 (19.8)	
Dabigatran, n (%)	14 (8.2)	5 (8.3)	9 (8.1)	
Rivaroxaban, n (%)	29 (17.0)	10 (16.7)	19 (17.1)	
**Concomitant antiplatelet therapy**				
SAPT + OAC, n (%)	29 (17.0)	11 (18.3)	18 (10.5)	0.73
Duration of SAPT + OAC, months	6 (2.5–12)	11 (3–12)	5 (1.75–8.3)	0.19
DAPT + OAC, n (%)	25 (14.6)	1 (1.7)	24 (21.6)	<0.0001
Duration of DAPT + OAC, months	3 (1.5–3)	0	3 (2–3)	0.16
**HFrEF, N (%)**	102 (59.6)	30 (50.0)	72 (64.9)	0.06
**Echocardiographic findings**				
Initial ejection fraction, %	34 ± 11	34 ± 12	34 ± 11	0.98
Initial WMSI, n	2.0 ± 0.3	2.1 ± 0.3	2.0 ± 0.4	0.02
Follow up ejection fraction, %	37 ± 12	37 ± 11	37 ± 13	0.24
Follow up WMSI, n	2.0 ± 0.4	2.1 ± 0.4	1.9 ± 0.4	0.10

Values are mean ± SD if normally distributed. *p*-values were calculated using the chi-square test for categorical variables and the Mann–Whitney test or Student’s *t*-test for continuous variables.

**Table 3:** Cox regression analysis identifying predictors of left ventricular thrombus resolution.

	Univariate		Multivariate	
Variables	HR (95% CI)	*p*-Value	HR (95% CI)	*p*-Value
DOAC	1.69 (1.13–2.53)	0.01	1.87 (1.01–3.45)	0.047
BSA	1.00 (0.92–1.10)	0.95	NA	NA
Age	0.99 (0.98–1.01)	0.34	0.99 (0.97–1.01)	0.28
Ischemic cardiomyopathy	0.76 (0.52–1.12)	0.16	NA	NA
STEMI at diagnosis	1.21 (0.83–1.76)	0.32	0.89 (0.49–1.59)	0.686
Atrial fibrillation	0.97 (0.59–1.57)	0.89	NA	NA
Concomitant antiplatelet therapy	1.86 (1.27–2.73)	0.002	2.37 (1.33–4.23)	0.003
Echocardiographic findings				
Initial ejection fraction	0.99 (0.97–1.00)	0.15	NA	NA
Initial WMSI	0.59 (0.31–1.10)	0.10	NA	NA
Final ejection fraction	1.00 (0.99–1.02)	0.74	NA	NA
Final WMSI	0.51 (0.26–0.99)	0.05	0.49 (0.25–0.97)	0.04

**Table 4:** Cardiovascular events in the overall study population and stratified by prescribed anticoagulant.

	DOAC (N = 99)			Warfarin (N = 72)			*p*-Value (Chi-Square Test)
Endpoints	N (%)	Patient/Years	New Cases/100 Pt Years	N (%)	Patient/Years	New Cases/100 Pt Years	
**Bleeding**							
Any	1 (1.0)	0.01	0.8	4 (5.6)	0.04	3.8	0.082
BARC ≥ 3	1 (1.0)	0.01	0.8	3 (4.2)	0.03	2.9	0.233
Blood transfusion	1 (1.0)	0.01	0.8	2 (2.8)	0.02	1.9	0.385
**Thromboembolic events, N (%)**							
Any, N (%)	3 (3.0)	0.02	2.4	6 (8.3)	0.06	5.7	0.125
Peripheral, N (%)	1 (1.0)	0.01	0.8	2 (2.8)	0.02	1.9	0.385
Stroke or TIA	2 (2.0)	0.02	1.6	4 (5.6)	0.04	3.8	0.109
**Death**	5 (5.1)	0.04	4.0	4 (5.6)	0.04	3.8	0.085

## Data Availability

The data that support the findings of this study are available upon request.

## Supplementary Materials

The following supporting information can be downloaded at: https://www.mdpi.com/article/10.3390/jcm14062129/s1, Table S1: Results of Cox Proportional Hazard Regression Analysis for interaction. Figure S1. DOAC distribution according to dose reduction criteria.

## List of Abbreviations

APT	Antiplatelet therapy
BARC	Bleeding Academic Research Consortium
BSA	Body surface area
CMR	Cardiac magnetic resonance
CT	Computed tomography
DAPT	Double antiplatelet therapy
DOAC	Direct oral anticoagulants
HfrEF	Heart failure with reduced ejection fraction
HTx	Heart transplantation
INR	International normalized ratio
IQR	Interquartile range
LMWH	Low-molecular-weight heparin
LV	Left ventricle
LVAD	Left ventricle assist device
LVT	Left ventricular thrombus
MACE	Major adverse cardiovascular event
MI	Myocardial infarction
PCI	Percutaneous coronary intervention
PT	Patient
SAPT	Single antiplatelet therapy
SD	Standard deviation
STEMI	ST-elevation myocardial infarction
TDVi	Telediastolic indexed volume
TTE	Transthoracic echocardiography
UFH	Unfractionated heparin
VKA	Vitamin K antagonist
WMSI	Wall motion severity index

## References

[1] Johannessen K.A., Nordrehaug J.E., von der Lippe G. (1984). Left ventricular thrombosis and cerebrovascular accident in acute myocardial infarction. Heart.

[2] Solheim S., Seljeflot I., Lunde K., Bjørnerheim R., Aakhus S., Forfang K., Arnesen H. (2010). Frequency of Left Ventricular Thrombus in Patients with Anterior Wall Acute Myocardial Infarction Treated with Percutaneous Coronary Intervention and Dual Antiplatelet Therapy. Am. J. Cardiol..

[3] Adams P.C., Cohen M., Chesebro J.H., Fuster V. (1986). Thrombosis and embolism from cardiac chambers and infected valves. J. Am. Coll. Cardiol..

[4] Kleindorfer D.O., Towfighi A., Chaturvedi S., Cockroft K.M., Gutierrez J., Lombardi-Hill D., Kamel H., Kernan W.N., Kittner S.J., Leira E.C. (2021). 2021 Guideline for the Prevention of Stroke in Patients with Stroke and Transient Ischemic Attack: A Guideline From the American Heart Association/American Stroke Association. Stroke.

[5] Byrne R.A., Rossello X., Coughlan J.J., Barbato E., Berry C., Chieffo A., Claeys M.J., Dan G.-A., Dweck M.R., Galbraith M. (2023). 2023 ESC Guidelines for the management of acute coronary syndromes: Developed by the task force on the management of acute coronary syndromes of the European Society of Cardiology (ESC). Eur. Heart J..

[6] Makam R.C.P., Hoaglin D.C., McManus D.D., Wang V., Gore J.M., Spencer F.A., Pradhan R., Tran H., Yu H., Goldberg R.J. (2018). Efficacy and safety of direct oral anticoagulants approved for cardiovascular indications: Systematic review and meta-analysis. PLoS ONE.

[7] Abdelnabi M., Saleh Y., Fareed A., Nossikof A., Wang L., Morsi M., Eshak N., Abdelkarim O., Badran H., Almaghraby A. (2021). Comparative Study of Oral Anticoagulation in Left Ventricular Thrombi (No-LVT Trial). J. Am. Coll. Cardiol..

[8] Alcalai R., Butnaru A., Moravsky G., Yagel O., Rashad R., Ibrahimli M., Planer D., Amir O., Elbaz-Greener G., Leibowitz D. (2022). Apixaban vs. warfarin in patients with left ventricular thrombus: A prospective multicentre randomized clinical trial. Eur. Heart J. Cardiovasc. Pharmacother..

[9] Isa W.Y.H.W., Hwong N., Yusof A.K.M., Yusof Z., Loong N.S., Wan-Arfah N., Naing N.N. (2020). Apixaban versus Warfarin in Patients with Left Ventricular Thrombus: A Pilot Prospective Randomized Outcome Blinded Study Investigating Size Reduction or Resolution of Left Ventricular Thrombus. J. Clin. Prev. Cardiol..

[10] Youssef A.A., Alrefae M.A., Khalil H.H., Abdullah H.I., Khalifa Z.S., Al Shaban A.A., Wali H.A., AlRajab M.R., Saleh O.M., Nashy B.N. (2023). Apixaban in Patients with Post-Myocardial Infarction Left Ventricular Thrombus: A Randomized Clinical Trial. CJC Open.

[11] Von Elm E., Altman D.G., Egger M., Pocock S.J., Gøtzsche P.C., Vandenbroucke J.P. (2008). The Strengthening the Reporting of Observational Studies in Epidemiology (STROBE) statement: Guidelines for reporting observational studies. J. Clin. Epidemiol..

[12] Mehran R., Rao S.V., Bhatt D.L., Gibson C.M., Caixeta A., Eikelboom J., Kaul S., Wiviott S.D., Menon V., Nikolsky E. (2011). Standardized Bleeding Definitions for Cardiovascular Clinical Trials: A consensus report from the bleeding academic research consortium. Circulation.

[13] Zhang Q., Zhang Z., Zheng H., Qu M., Li S., Yang P., Si D., Zhang W. (2022). Rivaroxaban in heart failure patients with left ventricular thrombus: A retrospective study. Front. Pharmacol..

[14] Jones D.A., Wright P., Alizadeh M.A., Fhadil S., Rathod K.S., Guttmann O., Knight C., Timmis A., Baumbach A., Wragg A. (2021). The use of novel oral anticoagulants compared to vitamin K antagonists (warfarin) in patients with left ventricular thrombus after acute myocardial infarction. Eur. Heart J. Cardiovasc. Pharmacother..

[15] Zhou Y., Zhang X., Lin Y., Peng W. (2024). Direct oral anticoagulants compared with warfarin in patients with left ventricular thrombus: A cohort study from China. J. Thorac. Dis..

[16] Al-Abcha A., Clay S., Wang L., Prasad R.M., Salam M.F., Srivastava S., Boumegouas M., Abela G.S., Saleh Y., Essa E.M. (2025). Direct Oral Anticoagulants Versus Warfarin for the Treatment of Left Ventricular Thrombus: A Multicenter Retrospective Observational Study. Am. J. Cardiol..

[17] Wiggins B.S., Dixon D.L., Neyens R.R., Page R.L., Gluckman T.J. (2020). Select Drug-Drug Interactions with Direct Oral Anticoagulants: JACC Review Topic of the Week. J. Am. Coll. Cardiol..

[18] Lattuca B., Bouziri N., Kerneis M., Portal J.-J., Zhou J., Hauguel-Moreau M., Mameri A., Zeitouni M., Guedeney P., Hammoudi N. (2020). Antithrombotic Therapy for Patients with Left Ventricular Mural Thrombus. J. Am. Coll. Cardiol..

[19] Lip G.Y., Gibbs C.R. (1999). Does heart failure confer a hypercoagulable state? Virchow’s triad revisited. J. Am.Coll. Cardiol..

[20] Guddeti R.R., Anwar M., Walters R.W., Apala D., Pajjuru V., Kousa O., Gujjula N.R., Alla V.M. (2020). Treatment of Left Ventricular Thrombus with Direct Oral Anticoagulants: A Retrospective Observational Study. Am. J. Med..

[21] McCarthy C.P., Murphy S., Venkateswaran R.V., Singh A., Chang L.L., Joice M.G., Rivero J.M., Vaduganathan M., Januzzi J.L., Bhatt D.L. (2019). Left Ventricular Thrombus: Contemporary Etiologies, Treatment Strategies, and Outcomes. J. Am. Coll. Cardiol..

[22] Fleddermann A.M., Hayes C.H., Magalski A., Main M.L. (2019). Efficacy of Direct Acting Oral Anticoagulants in Treatment of Left Ventricular Thrombus. Am. J. Cardiol..

[23] Robinson A.A., Trankle C.R., Eubanks G., Schumann C., Thompson P., Wallace R.L., Gottiparthi S., Ruth B., Kramer C.M., Salerno M. (2020). Off-label Use of Direct Oral Anticoagulants Compared with Warfarin for Left Ventricular Thrombi. JAMA Cardiol..

[24] Ali Z., Isom N., Dalia T., Sami F., Mahmood U., Shah Z., Gupta K. (2020). Direct oral anticoagulant use in left ventricular thrombus. Thromb. J..

[25] Iqbal H., Straw S., Craven T.P., Stirling K., Wheatcroft S.B., Witte K.K. (2020). Direct oral anticoagulants compared to vitamin K antagonist for the management of left ventricular thrombus. ESC Heart Fail..

[26] Daher J., Da Costa A., Hilaire C., Ferreira T., Pierrard R., Guichard J.B., Romeyer C., Isaaz K. (2020). Management of Left Ventricular Thrombi with Direct Oral Anticoagulants: Retrospective Comparative Study with Vitamin K Antagonists. Clin. Drug Investig..

[27] Cochran J.M., Jia X., Kaczmarek J., Staggers K.A., Al Rifai M., Hamzeh I.R., Birnbaum Y. (2021). Direct Oral Anticoagulants in the Treatment of Left Ventricular Thrombus: A Retrospective, Multicenter Study and Meta-Analysis of Existing Data. J. Cardiovasc. Pharmacol. Ther..

[28] Camaj A., Fuster V., Giustino G., Bienstock S.W., Sternheim D., Mehran R., Dangas G.D., Kini A., Sharma S.K., Halperin J. (2022). Left Ventricular Thrombus Following Acute Myocardial Infarction: JACC State-of-the-Art Review. J. Am. Coll. Cardiol..

[29] Weinsaft J.W., Kim J., Medicherla C.B., Ma C.L., Codella N.C.F., Kukar N., Alaref S., Kim R.J., Devereux R.B. (2016). Echocardiographic Algorithm for Post–Myocardial Infarction LV Thrombus: A Gatekeeper for Thrombus Evaluation by Delayed Enhancement CMR. JACC Cardiovasc. Imaging.

