# Supplementary material for: The Efficacy and Safety of Direct Oral Anticoagulants Compared to Warfarin for Left Ventricular Thrombus Resolution
Source: J Clin Med. 2025 Mar 20;14(6):2129. doi: 10.3390/jcm14062129 (PMC11942919; doi:10.3390/jcm14062129)
Supplement: Supplementary file 1 [file jcm-14-02129-s001.zip › jcm-3517319-Supplementary.pdf]

Supplements

Table S1. Results of Cox Proportional Hazard Regression Analysis for interaction.

	Univariate	
**Variables**	**HR (95% CI)**	**P Value**
**DOAC**	2.781 (1.280-6.044)	0.010
**Concomitant antiplatelet therapy**	3.000 (1.377-6.535)	0.006
**DOAC x Concomitant antiplatelet therapy**	0.646 (0.261-1.602)	0.346

DOAC stands for direct oral anticoagulant.

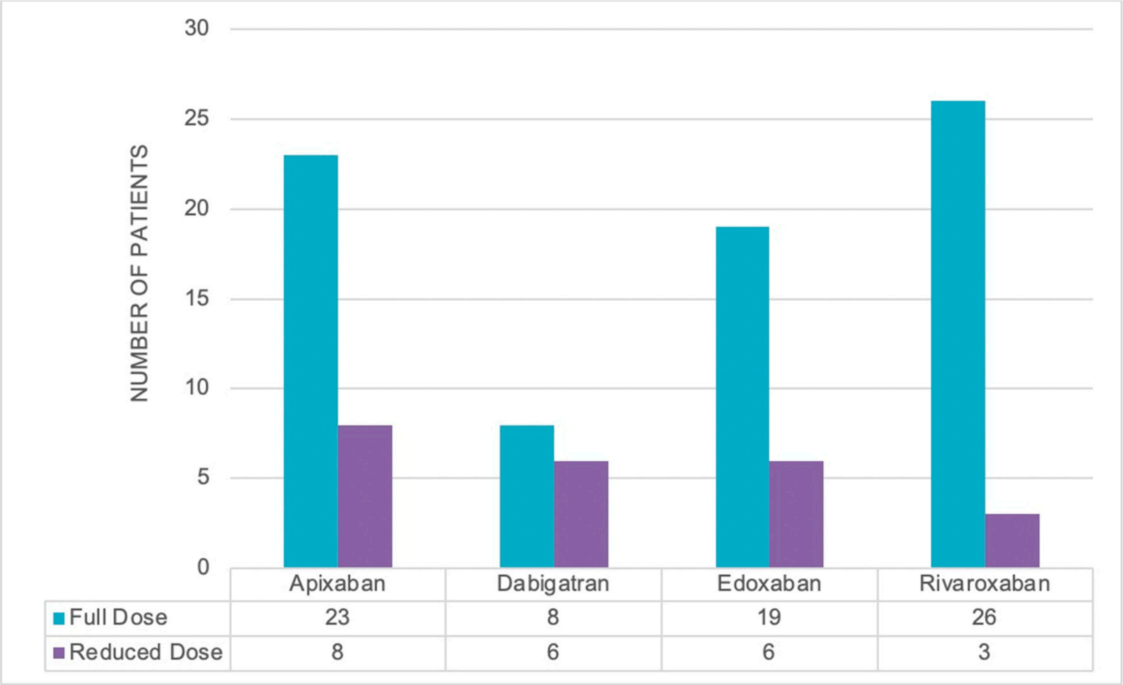

Figure S1. DOAC distribution according to dose reduction criteria.
